# Supplementary material for: Complexome profiling of the Chlamydomonas psb28 mutant reveals TEF5 as an early PSII assembly factor
Source: Plant Cell. 2025 Mar 17;37(6):koaf055. doi: 10.1093/plcell/koaf055 (PMC12164590; doi:10.1093/plcell/koaf055)
Supplement: koaf055_Supplementary_Data [file koaf055_supplementary_data.zip › psb28_interactive.zip!/plots/intensity/qualityPlots/peptides/psbA_Cre-1.g2716987.t1.p1.html]

### Cre-1.g2716987.t1.p1

Trivial name: psbA  
SeqCoverage [median]: 30.10  
Mass: 39.04 kDa  
  
 

| Sequence | MissedCleavage | Mass [Da] | wtIntensity | muIntensity | Score |
| --- | --- | --- | --- | --- | --- |
| ANLGMEVMHER | 0 | 1285.59 | 1787328.16 | 276401.23 | 138.71 |
| ENSSLWAR | 0 | 961.46 | 94007.53 | 4165.28 | 150.05 |
| ETTENESANEGYR | 0 | 1498.62 | 1182942.25 | 148241.36 | 342.74 |
| ETTENESANEGYRFGQEEETYNIVAAHGYFGR | 1 | 3667.61 | 13441.74 | 0.00 | 59.87 |
| EWELSFR | 0 | 965.46 | 310629.08 | 0.00 | 190.71 |
| FGQEEETYNIVAAHGYFGR | 0 | 2187.00 | 658748.08 | 151459.47 | 241.79 |
| LIFQYASFNNSR | 0 | 1458.73 | 1215237.88 | 292326.04 | 250.92 |
| NAHNFPLDLASTNSSSNN | 0 | 1901.85 | 3212.12 | 0.00 | 136.30 |
| RENSSLWAR | 1 | 1117.56 | 19001.68 | 0.00 | 87.81 |
| TAILER | 0 | 701.41 | 493162.53 | 27983.89 | 159.19 |
| TAILERR | 1 | 857.51 | 332955.08 | 83597.08 | 166.17 |
| VLNTWADIINR | 0 | 1313.71 | 1227926.90 | 98870.29 | 297.90 |
|  |  |  | 7338593.0 | 1083044.6 |  |
